# Carotid intima-media thickness and cardiovascular risk in patients with diabetes mellitus type 2 and chronic kidney disease

**DOI:** 10.1080/0886022X.2020.1745237

**Published:** 2020-03-26

**Authors:** Tomoyuki Kawada

**Affiliations:** Department of Hygiene and Public Health, Nippon Medical School, Tokyo, Japan

I have read with great interest the paper entitled ‘Carotid intima-media thickness is an independent predictor of all-cause mortality and cardiovascular morbidity in patients with diabetes mellitus type 2 and chronic kidney disease’ by Roumeliotis et al. [1]. The authors conducted a prospective study to investigate the effect of intima-media-thickness of the carotid artery wall (cIMT) on cardiovascular (CV) morbidity and mortality in patients with chronic kidney disease (CKD) and diabetes mellitus type 2 (DM2). Increased body mass index (BMI), lower estimated glomerular filtration rate (eGFR) and male gender were significant predictors of increased cIMT. Hazard ratios (HRs) (95% confidence intervals [CIs]) of eGFR and cIMT for all-cause mortality were 0.96 (0.94–0.98) and 2.9 (1.03–7.99), respectively. In addition, HR (95% CI) of albuminuria and cIMT for future CV event were 1 (CI = 1.0–1.0) and 2.04 (1.1–3.78), respectively. I have some concerns on this study.

First, Cardoso et al. evaluated the effect of carotid atherosclerosis on CV event, all-cause mortality and renal outcome in patients with DM2 [2]. HR (95% CI) of 0.1 mm increment of common cIMT for CV event was 1.15 (1.02–1.31), and HR (95% CI) of increased carotid plaque score for renal outcome was 1.63 (1.09–2.43). In contrast, there was no significance in HR of IMT indicators for al-cause mortality. I suppose that the severity of diabetic nephropathy might affect CV event and all-cause mortality, and renal risk discrimination by cIMT should be specified by further study.

Secondly, during 7-year follow-up period, 44 patients died and 66 patients presented CV event. Events per independent variable in Cox proportional hazard models should be kept 10 or higher for stable estimation [3,4], and the authors used more than 10 independent variables for the prediction. I suppose that more evets are needed for risk assessment.

Finally, Okayama et al. conducted a retrospective cohort study to investigate factors for CV event in patients with DM2 [5]. Adjusted HRs (95% CIs) of cIMT change, baseline cIMT, smoking, eGFR, BMI and age for CV event were 2.24 (1.25–4.03), 1.02 (1.01–1.04), 2.21 (1.27–3.20), 0.98 (0.97–0.99), 1.11 (1.02–1.20) and 1.04 (1.01–1.08), respectively. They specified that cIMT progression and baseline cIMT were both significant predictors of CV event, and eGFR was also selected. This means that change of cIMT should be included for the risk assessment, and kidney dysfunction might also affect CV event.

Tomoyuki Kawada *Department of Hygiene and Public Health, Nippon Medical School, Tokyo, Japan* kawada@nms.ac.jp

## References

[1] Roumeliotis A, Roumeliotis S, Panagoutsos S, et al. Carotid intima-media thickness is an independent predictor of all-cause mortality and cardiovascular morbidity in patients with diabetes mellitus type 2 and chronic kidney disease. Ren Fail. 2019;41(1):131–138. 30909780 10.1080/0886022X.2019.1585372 PMC6442115

[2] Cardoso CRL, Salles GC, Leite NC, et al. Prognostic impact of carotid intima-media thickness and carotid plaques on the development of micro- and macrovascular complications in individuals with type 2 diabetes: the Rio de Janeiro type 2 diabetes cohort study. Cardiovasc Diabetol. 2019;18(1):2. 30630491 10.1186/s12933-019-0809-1 PMC6327523

[3] Concato J, Peduzzi P, Holford TR, et al. Importance of events per independent variable in proportional hazards analysis. I. Background, goals, and general strategy. J Clin Epidemiol. 1995;48(12):1495–1501. 8543963 10.1016/0895-4356(95)00510-2

[4] Peduzzi P, Concato J, Feinstein AR, et al. Importance of events per independent variable in proportional hazards regression analysis. II. Accuracy and precision of regression estimates. J Clin Epidemiol. 1995;48(12):1503–1510. 8543964 10.1016/0895-4356(95)00048-8

[5] Okayama KI, Mita T, Gosho M, et al. Carotid intima-media thickness progression predicts cardiovascular events in Japanese patients with type 2 diabetes. Diabetes Res Clin Pract. 2013;101(3):286–292. 23835494 10.1016/j.diabres.2013.06.008

